# Drug–Drug Interactions Based on Pharmacogenetic Profile between Highly Active Antiretroviral Therapy and Antiblastic Chemotherapy in Cancer Patients with HIV Infection

**DOI:** 10.3389/fphar.2016.00071

**Published:** 2016-03-30

**Authors:** Massimiliano Berretta, Michele Caraglia, Ferdinando Martellotta, Silvia Zappavigna, Angela Lombardi, Carla Fierro, Luigi Atripaldi, Tommaso Muto, Daniela Valente, Paolo De Paoli, Umberto Tirelli, Raffaele Di Francia

**Affiliations:** ^1^Department of Medical Oncology, CRO National Cancer InstituteAviano, Italy; ^2^Department of Biochemistry, Biophysics and General Pathology, Second University of NaplesNaples, Italy; ^3^Hematology and Cellular Immunology (Clinical Biochemistry), A.O. dei Colli Monaldi HospitalNaples, Italy; ^4^Molecular Diagnostics Service, CETAC Research CenterCaserta, Italy; ^5^Hematology-Oncology and Stem Cell Transplantation Unit, National Cancer Institute, Fondazione “G. Pascale” IRCCSNaples, Italy

**Keywords:** pharmacogenomics, polymorphisms cytochrome P450, HIV, AIDS, antiretroviral therapy, cancer, antiblastic chemotherapy

## Abstract

The introduction of Highly Active Antiretroviral Therapy (HAART) into clinical practice has dramatically changed the natural approach of HIV-related cancers. Several studies have shown that intensive antiblastic chemotherapy (AC) is feasible in HIV-infected patients with cancer, and that the outcome is similar to that of HIV-negative patients receiving the same AC regimens. However, the concomitant use of HAART and AC can result in drug accumulation or possible toxicity with consequent decreased efficacy of one or both classes of drugs. In fact, many AC agents are preferentially metabolized by CYP450 and drug–drug interactions (DDIs) with HAART are common. Therefore, it is important that HIV patients with cancer in HAART receiving AC treatment at the same time receive an individualized cancer management plan based on their liver and renal functions, their level of bone marrow suppression, their mitochondrial dysfunction, and their genotype profile. The rationale of this review is to summarize the existing data on the impact of HAART on the clinical management of cancer patients with HIV/AIDS and DDIs between antiretrovirals and AC. In addition, in order to maximize the efficacy of antiblastic therapy and minimize the risk of drug–drug interaction, a useful list of pharmacogenomic markers is provided.

## Introduction

The assessment of Highly Active Antiretroviral Therapy (HAART) into the clinical setting had a striking impact on the clinical outcome of HIV-related cancers. The range of cancers diagnosed among patients infected by HIV/AIDS includes AIDS-defining diseases (ADC—Kaposi's sarcoma and non-Hodgkin's lymphoma) and non-AIDS-defining cancers (NADC—Hodgkin's disease, invasive anal carcinoma, lung carcinoma, skin cancer, colorectal cancer, and hepatocellular carcinoma). In fact, after the introduction of HAART, a decrease in ADCs and an increase in NADCs were observed due to the aging of HIV-positive cancer patients (Antoniou and Tseng, 2005). The challenge in the treatment of HIV-related diseases is the need to maintain an adequate management of HIV infection during the antiblastic chemotherapy (AC; Vaccher et al., 2001). AC induces a significant decrease in the number of CD4 lymphocytes and significantly increases the risk of opportunistic infections (OIs) in patients with HIV-related malignancies. Patients who receive a combination of AC and HAART can achieve better response and survival rates than patients who receive AC alone. The combined treatment is feasible and reduces the incidence of OI complications. However, careful attention must be paid to cross toxicity and possible pharmacokinetic and pharmacodynamic interactions between antiretrovirals and AC. Drug–drug interactions (DDIs) occur when one drug influences the level or activity of another when concurrently administered. They may result in increased therapeutic or adverse events, decreased therapeutic or toxicity or a single response that does not occur when either agent is administered alone (Mounier et al., 2009)

DDIs could be arise at all levels and a failure to identify them can result in overdosing or under-dosing the patient. DDIs are a primary concern in treatment and are more prevalent in the field of oncology. This could be due to the narrow therapeutic index and the inherent toxicity of AC. The risk of DDIs has been found to increase with the number of simultaneous medications. Cancer patients receive many drugs during their therapies including those for comorbidity and cancer-related symptoms such as pain, depression, and emesis. Therefore, they are at augmented risk to develop DDIs. According to Corona and colleagues, DDIs are frequent in oncology (Corona et al., 2008). In most cases, the consequences of DDIs are unwanted, compromising the effectiveness of the therapeutic agents or enhancing their toxicity. It has been reported that about 20–30% of all adverse drug events are caused by interactions between medications. To date few partial data are available on DDIs in the treatment of HIV-associated cancers. Protease inhibitors (PIs) and non-nucleoside reverse transcriptase inhibitors (NNRTIs) are potent inhibitors/inducers of the cytochrome P450 (CYP450) metabolic system. Since many AC are also metabolized by the CYP450 system, co-administration with HAART could result in drug piling up and possible adverse event or decrease the efficacy of one or both type of drugs (Dubrow et al., 2012).

In this field, the inter-individual response could be dependent on genetic variability in the population (Di Francia et al., 2015a). A few examples showing the correlation between toxicities and single nucleotide polymorphisms (SNPs) in the genes coding for metabolizing enzymes and drug-transporters are described here.

Amplified toxicity may lead to a delay of chemotherapy recycling or to a prompt dose reduction, possibly compromising the therapeutic benefit of AC (Flepisi et al., 2014). Toxicity can also negatively affect antiretroviral therapy compliance, favoring the emergence of resistant HIV strains. Recent data have shown that toxicity, particularly myelosuppression, and neurotoxicity, is significantly more common in patients treated with combined therapy than in patients treated with antineoplastic drugs alone (Harrys and Mulanovich, 2014). Alternatively, patients treated with chemotherapy plus HAART have a better survival rate than patients treated with chemotherapy alone, suggesting that the reduction of OI morbidity caused by HAART with the consequent amelioration of their performance status, can improve the overall outcome in the combined treatment setting (Beumer et al., 2014). This paper reviews the potential interactions and subsequent therapeutic considerations in the combination of HAART and AC used in the treatment of HIV-positive cancer patients.

## Haart classification and drug metabolism

In general, guidelines for naive HIV patients recommend the combination of three active drugs in order to prevent the occurrence of resistance: a combination of two nucleoside reverse transcriptase inhibitors (NRTIs) with either an NNRTI, or a PI boosted with ritonavir, or an integrase strand transfer inhibitor (INSTI). US Department of Health and Human Services (DHHS) guidelines recommend HAART for all HIV-1 patients with a CD4 T cell count <500 cells/μL, in order to preserve immune function while declining HIV-associated comorbidity and mortality. Similar regimens can be used in HIV-positive cancer patients according to the treatment plan (AC or radiotherapy or surgery), the presence of liver or renal diseases, bone marrow suppression, mitochondrial dysfunction, and patient preference (Harrys and Mulanovich, 2014). For the majority of antiretroviral drugs that are CYP 450 substrates, inducers or inhibitors, co-administration with other CYP 450 metabolized drugs can result in drug accumulation and potential toxicity or decreased efficacy of one or multidrugs (Rudek et al., 2011; Beumer et al., 2014). Particularly, inhibitor drugs for CYP450 enzymes typically cause reduced metabolism of other drugs that are substrate of the same enzyme (Table 1). This decreased metabolism may result in higher plasma drug levels and increased toxicity. Inhibition of CYP450 is rapid, with the maximal inhibitory effect going up when steady-state concentrations of the inhibitor are recognized. Equally, induction of the CYP450 system results in the augmented clearance of drugs concurrently metabolized by the same enzyme and a decrease of the drug concentration. Enzyme induction occurs more gradually than inhibition because the complete effect of the drug depends on the time necessary for new enzyme creation and the half-life of the inducing molecules (Mounier et al., 2009).

**Table 1:** **Summary of Pharmacokinetic characteristics of HAART**.

**HAART drug class**	**Metabolic fate**	**Hepatic inhibitors**	**Hepatic inducer**	**Pharmacogenomics evidences**
NRTIs	Several nucleosidase	No evidence	No evidence	Screening for HLA-B*5701 For prevention of the hypersensitivity to Abacavir
	Abacavir, Tenofovir			Tenofovir Nephro-Toxicity in carriers of ABCC4 (3436GG)
NNRTIs	Efavirenz, nevirapine: CYP3A4, CYP2B6 (minor)	Efavirenz: CYP2C9, CYP2C19	Efavirenz: CYP3A4 (potent), CYP2B6*22, UGT1A1	Nevirapine and Efavirenz neurotoxicity in CYP2B6*6 (516G>T) homozygous individuals
	Etravirine: CYP3A4, CYP2C9, and CYP2C19	Etravirine: CYP2C9 (weak), CYP2C19 (moderate), P-gp (weak)	Etravirine: CYP3A4 (weak)	Nevirapine hepatotoxicity in polymorphic ABCB1 3435CC allele (MDR1)
	Rilpivirine: CYP3A4 (major), as well as CYP2C19, 1A2, 2C8/9/10 (minor)	Delavirdine; CYP3A4 (potent)	Nevirapine: CYP3A4, CYP2B6 (potent)	
			Rilpivirine: CYP2C19 (moderate), CYP1A2, CYP2B6, and CYP3A4 (weak). A clinically relevant effect on CYP enzyme activity is considered unlikely with the 25 mg dose	
PIs	Mainly CYP3A4 and UGT1A1/3	darunavir, indinavir, nelfinavir, amprenavir >> saquinavir	Nelfinavir: UGT, CYP2B6, CYP2C8, CYP2C9/19	Favorable response (in term of viral suppression) to Nelfinavir in CYP2C19*2 and *3 poor metabolizer patients
		Atazanavir: 3A4, UGT1A1 >> 2C8 (weak)	Ritonavir: UGT, CYP1A2, CYP2C9/19, CYP2B6	Decreased clearance of, indinavir and saquinavir in haplotype CYP3A5*3. Lopinavir plasma levels are higher in SLCO1B1 521CC allele than 521TT
		Caution when unboosted atazanavir is coadministered with drugs that are CYP2C8 substrates with narrow therapeutic indices (e.g., paclitaxel, repaglinide); clinically significant interactions with CYP2C8 substrates are not expected when atazanavir is boosted with ritonavir	Tipranavir: mixed induction/inhibition effects; often acts as inducer of CYP3A4 (potent) and UGT, even when boosted with ritonavir	High level of bilirubinemia among patients homozygous for the UGT1A1*28 for indinavir and Atazanavir administration
		Nelfinavir: CYP2B6 *in vitro*		
		Ritonavir: CYP3A4 (potent) >>2D6 > 2C9 > 2C19 > 2A6 > 1A2 > 2E1		
		Atsmall boosting doses, ritonavir has a negligible		
		The effect of CYP2D6 inhibition		
		Ritonavir inhibits CYP2B6 *in vitro* but induces CYP2B6 *in vivo*		
		Tipranavir: CYP2D6		
INSTIs	Dolutegravir: UGT1A1, CYP3A4 (10–15%)	Cobicistat: CYP3A, CYP2D6; also P-glycoprotein	Dolutegravir does not induce CYP1A2, CYP2B6, or CYP3A4 *in vitro*	
	Elvitegravir: CYP3A, UGT1A1/3	(P-gp), BCRP, OATP1B1, and OATP1B3	Elvitegravir: CYP2C9 (modest)	
	Cobicistat: CYP3A, CYP2D6 (minor)		Dolutegravir inhibits the renal organic cation transporter, OCT2	
		Raltegravir: UGT1A1		
CCR5 receptor antagonists	Maraviroc: CYP3A family	No evidence	No evidence	evidence of increased risk of susceptibility to hepatitis C virus infection or multiple sclerosis among individuals with CCR5-delta32 mutation
Fusion inhibitors	Enfuvirtide is ligand for viral gp41	No evidence	No evidence	

*ABC, ATP binding cassette; MDR, multidrug resistance; MRP, multidrug resistance-associated protein; OAT, organic anion transporter; OATP, organic anion-transporting polypeptide; OCT, organic cation transporter; OCTN, organic cation/carnitine transporter, novel type; SLC, solute carrier; SLCO, solute carrier organic anion*.

### Nucleoside reverse transcriptase inhibitors (NRTIs)

Nucleoside reverse transcriptase inhibitors (NRTIs), sometimes called “nucleoside analogs” or “nukes,” contain faulty versions of the building blocks (nucleotides) used by reverse transcriptase (RT). The RT enzyme has two enzymatic functions. Firstly, it acts as a where it transcribes the single-stranded into single-stranded DNA and subsequently it builds a complementary strand of DNA. This provides a DNA double helix which can be integrated into the host cell's chromosomes. Secondly, it has ribonuclease activity as it degrades the RNA strand of the RNA–DNA intermediate that is formed during viral *de novo* DNA synthesis. RT incorporates the faulty NRTI building blocks and *de novo* DNA cannot be correctly synthesized. As a result, HIV's genes can't be incorporated into the healthy *de novo* DNA and the cell cannot produce new viruses. For NRTIs, probability for DDIs is minimal because these agents are not eliminated by the CYP 450 system and do not induce or inhibit CYP 450 enzymes. However, NRTIs may be victims of transporter-mediated interactions because renal clearance is their primary route of elimination. NRTI-based treatments are associated with anemia, dyslipidemia, diarrhea, emesis, insulin resistance, neutropenia, nephrotoxicity, lactic acidosis, hepatosteatosis, and an improved risk of cardiovascular adverse effects (Harrys and Mulanovich, 2014). Tenofovir may lead to renal dysfunction principally in patients getting nephrotoxic drugs. Renal function must be monitored over time, and the dose adjusted in the case of nephropathies. Patients under treatment with abacavir (ABC)-lamivudine in predetermined dose combination, genetic screening for HLA-B* 57.01 should be performed to prevent the risk of a hypersensitivity reaction to ABC (Beumer et al., 2014). Susceptibility to this reaction appears to be genetic and has been associated with HLA-DR7 haplotypes. Recent data has shown a susceptibility locus within the B*57.01 haplotype that was present in 94% of patients with ABC hypersensitivity (Rudek et al., 2011).

### Non-nucleoside reverse transcriptase inhibitors (NNRTIs)

Non-nucleoside reverse transcriptase inhibitors (NNRTIs), despite their chemical diversity, bind all at the equivalent site in the RT. The binding occurs allosterically in a hydrophobic pocket located around 10 Å from the catalytic site of the p66 subunit of the enzyme. The NNRTI binding site (NNIBP) contains five aromatic, six hydrophobic, and five hydrophilic amino acids that belong to the p66 subunit and additional two amino acids (Ile-135 and Glu-138) belonging to the p51 subunit. Every NNRTI interacts with different aminoacid residues in the NNIBP, and all are extensively metabolized via the CYP450 enzyme system (Mounier et al., 2009). The probability for DDIs is elevated because these agents are widely metabolized by or inhibit the CYP450 system (Harrys and Mulanovich, 2014). These regimens are associated with rash, central nervous system toxicity, and high hepatic transaminase levels. Central Nervous System (CNS) side effects have been noted in up to 52% of patients but are sufficiently severe to require discontinuation in only 2 to 5%. There is a potential toxic additive effect with alcohol or other psychoactive drugs. Nevirapine acts as an inducer of CYP3A4 and Efavirenz can either inhibit or induce CYP3A4 activity. Efavirenz most often acts as a CYP3A4 inducer and may also induce CYP2B6 (Tsuchiya et al., 2004). Etravirine, a second generation NNRTI, is a weak inducer of CYP3A and a weak inhibitor of P-glycoprotein and constitutes a valuable option for concomitant use with BEACOPP chemotherapy for advanced HD (Kurz et al., 2015). Rilpivirine is primarily metabolized by CYP3A but does not induce the P450 system and theoretically should not affect immunosuppressant drug levels (Tsuchiya et al., 2004).

### Protease inhibitors (PIs)

Protease Inhibitors (PIs), prevent viral replication by selectively binding to HIV-1 protease and blocking the production of infectious viral elements. The HIV protease contains a binding pocket into which drugs should fit to inhibit the activity of the enzyme. As HIV duplicates, constant mutations change the profile of this configuration. Drug resistance occurs when some of these mutations inhibit the binding of one or more PIs. The early resistance mutations that are selected can differ between PIs, but are all located near the substrate-binding gap of the enzyme. These primary mutations lead to simultaneous resistance to multiple PIs. During PI therapy, additional mutations (secondary mutations) should be identified in the protease that leads to high-level PI resistance. As a result, cross-resistance is one of the most important problems related with PI treatment (Shafer, 2006). Ritonavir (RTV) is one of the most powerful CYP3A4 inhibitor. Also, it is an active inhibitor of ABCB1, CYP2C8, CYP2D6 and a weak inducer of ABCB1, CYP2B6, CYP2C9, CYP3A4 (Kiser et al., 2008). Second-generation of PIs (i.e., atazanavir, darunavir, fosamprenavir, lopinavir, and tipranavir) are active against HIV acquired mutations because therapy with the older PIs. Darunavir and tipranavir are different from the others PIs in that they are synthetic non-peptide drugs. PI regimens are related with dyslipidemia, fat misdistribution, insulin resistance, hepatic transaminase elevation, and an increased risk of gastrointestinal and cardiovascular events. Hepatotoxicity is more frequent and more severe with full dose RTV than with other PIs. This toxicity is reduced with the lower RTV doses used in dual-PI combination. QT prolongation has been linked in particular with PIs such as atazanavir, saquinavir, and ritonavir-boosted lopinavir (Rudek et al., 2011)

### Integrase strand transfer inhibitors (INSTIs)

Integrase strand transfer inhibitors (INSTIs) are a class of antiretroviral drug aimed at blocking the action of integrase. Integrase are specialized viral enzyme able to inserts the viral genome into the DNA of the host cell. Since integration is a vital step in retroviral replication, blocking it can halt further spread of the virus. Since integrase inhibitors target a different step in the retroviral life cycle, they may be taken in combination with other types of HIV drugs to minimize adaptation by the virus. They are also useful in salvage therapy for patients whose virus has mutated and acquired resistance to other drugs. Raltegravir, the first approved INSTI, is metabolized only by UDP glucuronosyltransferase 1A1 (UGTA1A1) and is unlikely to have major interactions. Raltegravir is neither an inhibitor nor an inducer of CYP1A2, CYP2B6, CYP2C8, CYP2C9, CYP2C19, CYP2D6, and CYP3A. Also, it doesn't interfere with P-glycoprotein-mediated drug transportation. Therefore, it can be a suitable alternative for the prevention of AC–HAART interactions (Tsuchiya et al., 2004). It has been associated with abnormal creatine kinase plasma levels, rhabdomyolysis, and myopathy. Elvitegraviris is primarily metabolized by CYP3A4/5 and partly by glucuronidation via UGT1A1/3 and is a component of an FDA-approved fixed-dose combination tablet (Stribild) containing tenofovir alafenamide, emtricitabine, and the CYP3A4 inhibitor cobicistat. Dolutegravir has a minor role with CYP3A4 because it is metabolized by uridine 5-diphosphate-glucuronosyltransferase. Common side effects are insomnia, allergies, headache, and anomalous liver function in patients who have concurrent hepatitis B or C. Also, it causes augment of the serum creatinine level due to inhibition of tubular secretion of creatinine (Ritchie et al., 2006)

### CCR5 receptor antagonists

CCR5 receptor antagonists are a group of small molecules that blocking the CCR5 receptor. The C-C pattern chemokine receptors CCR5 and CXCR4 are the most important chemokine receptors implicated in the HIV entrance method. These receptors fit in to the seven transmembrane G-protein-coupled receptor' (GPCR) family and are mainly expressed on macrophages, T-cells, dendritic cells, and Langerhans cells. They cooperate as co-receptors that HIV-1 uses to bind cells before viral fusion and entry. HIV isolates could be divided into R5 and X4 strains, depending to the co-receptor used (respectively, CCR5 or CXCR4). The CCR5 antagonist maraviroc is a substrate of ABCB1 CYP3A but does not alter transport and/or metabolism. Also it is not likely to make DDIs. It has been related with upper respiratory tract infections, fever, rash, and hepatotoxicity (Ritchie et al., 2006).

### Fusion inhibitors

Fusion inhibitors are a class of molecules designed to disrupts the HIV-1 fusion protein equipment at the final stage of fusion with the hosting cell, and prevent non-infected cells from becoming infected. HIV binds to the host CD4+ cell receptor via the viral protein gp120; gp41, a viral transmembrane protein, and then undergoes a conformational change that assists in the fusion of the viral membrane with the host cell membrane. Enfuvirtide binds to gp41 preventing the formation of an entry pore for the capsid of the virus, keeping it out of the cell. Enfuvirtide is not metabolized by CYP450 but undergoes hydrolysis and to date no drug interaction have been noted with this agent. Side effects associated with enfuvirtide include diarrhea, fatig, nausea, and injection site reactions (Beumer et al., 2014).

## Role of pharmacogenomics associated with HAART

Even though the benefits of HAART, wide individual variability has been reported in reply to treatment and in the adverse effects of certain antiretroviral drugs. Indeed, response to HAART is extremely complex and often limited by the development of short- or long-term toxicities and the coming out of antiretroviral drug resistance. This unpredictability could be explained by factors that normalize the bio-availability of drugs (pharmacokinetics), special effects on the host (host pharmacodynamics), and the activity of the virus itself (viral pharmacodynamics).

It is clear that the efficacy of therapy depending by viral sensitivity to therapy. Mutagenesis in the viral genome is a physiological process. In fact, mutations should happen in each duplication cycle, so enabling the virus to adapt rapidly. Furthermore, initial antiretroviral therapy can be compromised by transmitted HIV drug resistance. A list of the primary viral resistance against HAART is available in Shafer (2006).

In addition to viral mutations, further factors may also provide to treatment failure, likely inter-individual variability in the pharmacokinetics of antiretroviral drugs. This may be direct, because sub-therapeutic drug concentration can augment the hazard of a low virologic response, or indirect, when high (toxic) drug levels lead notable intolerability, showing to poor adherence to the treatment. These (THIS) variability among patients is probably driven by genetic and environmental factors such as DDIs, body weight, drug–food interactions, and sex. In particular, DDIs and genetic polymorphisms in drug-metabolizing enzymes and drug transporters add to extensive variability in drug pharmacokinetics and toxicity. A few examples are reported in Table 1.

The *CYP2B6* gene is extremely polymorphic, and more than 28 alleles have been characterized (about 100 SNPs). Among different variants, the CYP2B6*6 haplotype (*516 G*>*T*, and *785A*>*G*) leads to reduced catalytic activity and a significant decrease in protein expression. Several studies have reported correlations of Nevirapine and Efavirenz to neurotoxicity with CYP2B6*6 (516G>T) homozygous individuals (Haas et al., 2005).

Several polymorphisms of the *CYP2C19* gene are associated with reduced enzyme activity. In particular, the CYP2C19*2 allele leads to a 681G>A substitution, causing a stop codon splicing variant. These poor metabolizing patients have a favorable response (in term of viral suppression) to exposure to Nelfinavir (Haas et al., 2005).

Unpredictability in metabolic CYP3A5 function is mainly accredited to the CYP3A5*3 polymorphic allele and, to a smaller extent, to the CYP3A5*6 and CYP3A5*7 variants. The variant CYP3A5*3 allele produces an alternative mRNA splicing, resulting in trunked protein due to the formation of a untimely stop codon (Anderson et al., 2009). Haplotype CYP3A5*3 has been related with significantly reduced clearance of both indinavir and saquinavir (Anderson et al., 2006).

The CCR5 antagonist maraviroc is a substrate of ABCB1 and CYP3A but does not alter drug transport or metabolism and is not likely to stimulate enzyme-mediated interactions. It has been associated with hepatotoxicity, and infections among individuals with CCR5-Delta32 mutations (Wheeler et al., 2007).

Association of polymorphisms in ATP-binding Cassette (*ABCC*) with efficacy of therapy was also found as drug transporters are seen as one of the primary mechanisms related to sub-therapeutic antiretroviral drugs concentrations of. Major studies shown a relationship between the *ABCB1* polymorphism (*3435 C*>*T*) and hepatotoxicity risk after nevirapine treatment. This genotype-phenotype association was established by Ritchie et al. (2006), who described that the *ABCB1 3435 TT* allele was fewer frequent in the patient group recording hepatic toxicity than polymorphic *3435CC*. Nevertheless, a pharmacogenetic study that integrated the *C421A* and *G34A* variants, that were linked *in vitro* with a decrease in ABCG2 activity, found no relationship of these polymorphisms with cellular accumulations of zidovudine and lamivudine triphosphate. Few studies are available on other Nucleosides analogs (Kohlrausch et al., 2010).

Recent data propose an important role for influx via the Solute Carrier Organic Transporters (SLCO alias OATP) family in the pharmacokinetics of antiretroviral agents. In detail, it has been observed that the *SLCO1B1 521T*>*C* polymorphism was significantly related to higher plasma concentrations of lopinavir in patients homozygous for the mutant allele (*521CC*), which would suggest that the entry of lopinavir into the liver via the SLCO1A2 influx transporter is an important determinant of exposure to lopinavir (Kohlrausch et al., 2010).

Recent studies in patients who received atazanavir and indinavir established that the proportion of grade 3–4 hyperbilirubinemia was 80% among patients homozygous for the UGT1A1*28 allele, 29% in heterozygous patients and 18% in patients homozygous for the wild-type allele, respectively (Rodríguez-No'voa, S. et al, 2007).

Even if there is clinical usefulness of the described polymorphisms implicated in HAART based-therapy, whether pharmacogenetics testing improves clinical outcomes is still an open query. In fact, the cost-effectiveness of the genotyping is still unknown and clinical expertise in the interpretation of laboratory results is urgently needed (Di Francia et al., 2012, 2014).

## Assessing hepatic function in patients on HAART

Bilirubin values is frequently used as a guide for dosage adjustment in AC. Several antiretrovirals such as atazanavir or indinavir are related with unconjugated hyperbilirubinemia resulting to UGT1A1 inhibition similarly to that occurring in Gilbert's syndrome. Unconjugated hyperbilirubinemia in combination with HAART and in the absence of other evidence of hepatic dysfunction could be unnoticed in AC dosing. Conversely, NRTIs such as didanosine, stavudine, and zidovudine can produce steatosis and lactic acidosis. These antiretrovirals should be blocked or replaced prior to initiating AC agents that undergo hepatic metabolism. In fact, NRTIs such as abacavir, emtricitabine, lamivudine, and tenofovir or NNRTIs such as efavirenz are fewer probable to be hepatotoxic and could be used in place of the others (Rudek et al., 2011). In addition, several cases have been associated to primary hepatocellular carcinoma in HIV-positive patients (Nunnari et al., 2012).

## Anticancer treatment overview

The therapeutic strategy should take into consideration three essential elements: (i) the histological nature of the neoplasm; (ii) the estimation of the extension of the tumor disease; (iii) the evaluation of the general state of disease. Surgery and/or radiation therapy provide local control of the tumor while AC is dedicated to the prevention or treatment of metastatic disease. In recent years, monoclonal antibodies have achieved a great clinical diffusion and therefore, the importance of both the identification of tumor targets and the characterization of the resistance mechanisms is increasing (Shafer, 2006).

### Kaposi's sarcoma (KS)

In the HAART era, KS remains the second most frequent tumor in HIV-infected patients worldwide (Martellotta et al., 2009). Treatment decisions have to take into consideration the extent and the rate of tumor growth, the patient's symptoms, immune system conditions, concurrent HIV-related complications, and Epstein–Barr virus (EBV) co-infection (Pinzone et al., 2015). Local therapy (intralesional vinblastine, oral etoposide, cryotherapy, and excisional surgery) is reserved for patients with minimum skin disease as cosmetics, and as palliative treatment for non-responders to systemic AC who have speedily progressive disease (PD; Simonelli et al., 2009). Radiotherapy is efficacy and usually represents the best topic therapeutic approach for treatment of pain, edema, and bleeding. HAART including PI, alone or in combination with local therapy, represent the first-line treatment for stage T0 and T1 slowly PD. HAART with concomitant AC is eligible for visceral disease and/or rapidly progressive disease, and maintenance (M)-HAART after AC can be useful as anti-KS treatment after AC (91% overall response rate). Systemic AC is reserved for non-responders patients to HAART and/or have widespread, symptomatic, rapidly PD, life-threatening disease with visceral involvement and an IRIS-associated flare. Liposomal anthracyclines (doxorubicin 20 mg/m^2^ i.v. every 2 weeks or daunorubicin citrate liposome 40 mg/m^2^ i.v. every 2 weeks) are now considered as the first-line therapy for patients with advanced AIDS-KS. Intravenous paclitaxel (100 mg/m^2^ given every 2 weeks as a 3-h infusion) or intravenous Irinotecan (150 mg/m^2^ day 1; 10 every 21 days) plus HAART including PI is reserved for patients with persistent or recurrent AIDS-related KS after than first-line AC (Corona et al., 2008).

### Non-hodgkin's lymphoma (NHL)

From the time when the beginning of the AIDS epidemic NHL has been related with HIV infections. About 80–90% of HIV-associated NHLs are classified as intermediate or high grade NHL, and almost all are of B lymphocyte origin. The development of HIV-associated NHL has been shown to be connected to older age (>35 years), low CD4 cell count (<100/mm^3^), high serum LDH, and no previous treatment with HAART. In addition, these factors jointly with low performance status (>2) and the involvement of more than two extra nodal sites are related with poor clinical outcome and shorter survival in HIV-NHL. It is also contemplation that immune stimulation by HIV and reactivation of prior EBV infection owing to imperfect T-cell surveillance leads to long-term stimulation and proliferation of B-lymphocytes, resulting in the development of HIV-NHL (7). Since the extensive use of HAART, the prognosis of HIV-NHL has improved, with a better tolerance to AC, a higher complete remission (CR) rate, a significant improvement in disease-free survival (DFS) and an important reduction in the number of deaths related to HIV complications. Many studies have shown that the R-CHOP regimen (rituximab plus cyclophosphamide, doxorubicin, vincristine, and prednisone) can be considered as the conventional approach for patients with CD20-positive diffuse large B-cell NHL in the HIV setting (Vaccher et al., 2001). With respect to the make use of rituximab, it is significant to point out contradictory results. Recent results have emphasized reservations on the safety of the R-CHOP combination therapy in patients with HIV (Beumer et al., 2014).

#### Hodgkin's lymphoma (HD)

It is described that HIV-infection increases the risk of developing HD about 8- to 10-fold compared with the healthy population (Rios, 2014). HIV-HD is characterized by numerous critical features such as elevated frequency of advanced stage disease (i.e., mixed cellularity or lymphocyte depleted histological subtypes), and extra nodal involvement. An optimal therapy for HIV-HD has not been defined (Rios, 2014). Since patients have advanced HD, they have been treated with polichemotherapy i.e., MOPP (mechlorethamine, vincristine, procarbazine, and prednisone), or currently with ABVD (doxorubicin, bleomycin, vinblastine, and dacarbazine), but the CR rate remains lower than that of “primary” HD. The main problem in therapy is immunosuppression caused by AC that can further synergize the immunocellular deficit of HIV-infected patients, and can facilitate the beginning of OIs and/or the evolution of the HIV infection itself. Also, even though CD4+ cell counts in these patients are typically normal or a little decreased at diagnosis, they may become strictly reduced during and after AC, leading to a higher susceptibility to OIs. In conclusion, leucopoenia, usually present in patients with HIV-HD due to previous therapy with nucleoside analogs and/or HIV-related myelodysplasia, sporadically makes conventional doses of AC difficult to administer. For the disseminated disease, early experience suggests that antiretrovirals can be used concurrently even with dose-intense regimens including the BEACOPP regimen (bleomycin, etoposide, doxorubicin, cyclophosphamide, vincristine, procarbazine, and prednisone; Cherif et al., 2015).

### Squamous cell carcinoma of the anus (SCCA)

The gastrointestinal (GI) tract is one of the most widespread sites of opportunistic infections in HIV-infected patients. SCCA is a relatively uncommon cancer of the GI tract, constituting only 1.5% of all digestive system cancers. Recent data describe not only a different SCCA incidence rate among HIV-positive and HIV-negative patients but also increased incidence rates of SCCA among HIV-infected patients in the HAART era compared to the pre-HAART era. The risk of SCCA is 120-fold greater in HIV-positive than in HIV-negative patients and in the setting of HIV it appears to be higher for patients with lower CD4+ T-cell counts. In addition to the augmented risk of developing SCCA, HIV-infected individuals may have higher SCCA-related morbidity and mortality (Martellotta et al., 2012). In fact, the 5-year survival ranges from 47 to 60%, which is lower than the 73% 5-year DFS rate reported in the common population. In relationship with HAART, a hard line approach to the treatment of SCCA in HIV-infected patients is necessary, as reported for other NADCs (Spina and Tirelli, 2004; Shafer, 2006). In fact, patients who receive the AC plus HAART combination can get the best response rates and the highest survival rates than those who receive AC alone. Surgery has been reserved for salvage therapy for non-responders or recurrent disease. A role for surgery also remains for selected patients with small, superficial tumors of the anal margin and those with lesions <2 cm that seldom have nodal involvement, and that can be advantaged by local excision. Several series and prospective trials have demonstrated the feasibility and efficacy of combined therapy consisting of radiotherapy (RT) and concurrent AC. This typically includes 5-fluorouracil (5 FU) and mitomycin C (MMC). Two European phase III randomized clinical trials evaluated the benefits of combined therapy vs. RT alone. Both trials demonstrated a significant increase in CR rates, an improvement in local control, and a significant decrease in local failure and need for colostomy in the combined therapy arm vs. the RT alone arm. For the combined therapy arm, a 5-year survival rate of 56% and 3-year survival rate of 65% were reported, respectively. An American phase III trial examined the importance of MMC in the standard combined therapy regimen and demonstrated a significant reduced local failure rate, and improved colostomy-free and DFS rate with the addition of MMC compared to 5-FU alone-based CMT (Zanet et al., 2011).

### Lung cancer

The increased incidence of lung cancer during the HAART era may be related to prolonged life expectancy of HIV-positive patients, the longer period of immune suppression of these patients and in particular the increased total number of cigarettes they smoke. Recent studies show that lung cancer is more common in association with acceptable immune competence than in the more advanced stages of HIV infection (Bearz et al., 2014). Because lung cancer has an impact on the overall survival (OS) of HIV-positive patients suffering from this neoplasia, the patients with advanced stage lung cancer should be treated accordingly to standard regimens used in the general population (platin-based chemotherapy and/or radiotherapy). The combination of AC and HAART is feasible and supports the protective effect of HAART which co-induces a significant improvement in the OS (Bearz et al., 2012).

### Cervical cancer

Invasive cervical cancer affects the uterine cervix. The mainly common histological types are squamous cell (69%) and adenocarcinoma (25%). It may be caused by a persistent Human Papillomavirus (HPV) infection; in particular HPV 16 and 18 are associated with more than 90% of cervical cancer cases. It has been known that women HIV patients have a higher incidence of HPV infection and the HPV–HIV co-infection is believed to induce both disruption and dysregulation of the humoral and cellular of local and systemic immunity with consequent rapid disease evolution. Generally, cervical intraepithelial neoplasia (CIN) progression forward to invasive cervical cancer in uninfected women can get some years those age between 45 and 50 years old. In women HIV patients, CIN progresses more speedily occurring between 16 and 40 years old and is usually resistant or responds less well to treatment (Dubrow et al., 2012).

### Colorectal cancer (CRC)

Colorectal cancer (CRC) is the third principal cause of cancer death in the general population. Considering that HIV is now a chronic disease, many patients are living long enough to develop CRC. However, few studies have evaluated the incidence of CRC in HIV-positive cohorts and the majority of them have not considered that the risk of CRC is increased among HIV infected people. However, a major limitation is the lack of data regarding rates of CRC screening in the HIV population due to scarce adherence to screening program. Available data suggest that HIV-infected patients with CRC present with more advanced disease and at a younger age than individuals without HIV infection (Di Benedetto et al., 2013). The GICAT group has compared the clinical presentation and outcome of 27 HIV-positive patients and 54 age- and sex-matched controls with CRC, concluding that HIV-positive patients had a lower PS, a poor Dukes' stage, a the highest grading and the shortest survival than uninfected subjects (Berretta et al., 2010). Regarding the concomitant use of HAART and AC, another study from the GICAT group has demonstrated that it is feasible, safe and effective, especially for the Folfox4 treatment in metastatic CRC HIV-positive patients (Berretta et al., 2008).

### Hepatocellular carcinoma (HCC)

Hepatocellular Carcinoma (HCC) is the most common primary cancer of the liver, and according to the WHO report, the fourth most common cause of death (Jong-wook, 2003; Bosch et al., 2004; Gomaa et al., 2008). The risk of HCC is seven-fold higher in HIV-infected than in HIV-uninfected patients. Since the introduction of HAART, no decrease in the incidence of HCC has been observed, unlike other HIV-associated cancers (Clifford et al., 2008; Berretta et al., 2011).

In the general population, HCC occurs several decades after the initial infection with HCV or HBV (El-Serag et al., 2008). Although, it was suspected that HIV infection alone may be a risk factor for HCC, this theory seems to have been excluded in huge retrospective cohort studies (Clifford et al., 2008). Conversely, in HIV-positive patients, co-infection with HCV or HBV is common and a notably higher risk of developing HCC through improvements in immune reconstitution due to chronic viral hepatitis are well-recognized. Still modest is the knowledge about the relations between HIV and HBV and/or HCV over the long-term: HIV co-infection seems to increase disease progression and decrease the efficacy of both anti-HCV and anti-HBV treatments. Nevertheless, it is ambiguous whether HIV infection directly increases the probability of HCC in viral hepatitis patients (Salmon et al., 2006; Berretta et al., 2011). In addition to the increased risk of rising HCC, patients with HIV infection may have higher HCC-related co-morbidity and death. Several studies have shown that HIV/HCV co-infected individuals with HCC, develop liver cirrhosis more quickly and more aggressive than HCV-mono-infected patients. However, the clinical procedures of HCC in an HIV-infected setting is not yet well-defined, since most earlier studies have had little sample sizes and/or many HIV patients were not undergoing HAART treatment.

Recently a GICAT Study, in a large retrospective analysis, demonstrated that in the majority cases of HCC-HIV patients, was diagnosed in patients with well-controlled HIV infection and a good PS (Berretta et al., 2011). The age at HCC diagnosis was younger in HIV-infected than in uninfected patients, and HCV co-infection was an additional risk factor, with a poorer prognosis in terms of lower median survival-time, when compared to HIV-uninfected patients, regardless of HIV-infection fine control. As predictable, HIV-patients also showed the shortest mean time of progression from Chronic Liver Disease to HCC.

Moreover, a lot of different significant findings come out from their results. First, HIV-infected patients that developed HCC were co-infected with HCV or HBV in the great greater part of cases. Second, in HIV-infected patients, HCC was diagnosed more frequently in the early stages (66% in stages A or B) and was for this reason amenable to curative approaches. In spite of this result, the median survival of the HIV-infected cohort was poorer with respect to that of the HIV-uninfected cohort, where HCC was diagnosed at more advanced stages (58% were in stages C or D) and was hence more infrequently amenable to curative approaches. Third, HIV-infected patients on HAART at HCC diagnosis showed a better prognosis than patients not on HAART. Fourth, no response rate differences were found as regards potentially curative treatments both at diagnosis and at recurrence between two groups, although in the case of HCC disease progression after treatment, HIV-infected patients were re-treated significantly less frequently than HIV-uninfected patients. The authors concluded that in their study HCC-HIV-positive patients showed a significant higher median survival (35 months) with respect to data reported by a previous study (Di Benedetto et al., 2013). Additional significant data emerging from the GICAT study concerning the treatment scheduled for HIV-infected patients: in about one-third of HIV patients, HCC was treated with potentially curative options. More recently, also from the GICAT group, it has been demonstrated for the first time that the concomitant use of Sorafenib and HAART is safe and feasible, without major complications and/or toxicities reported (Berretta et al., 2013). In conclusion the concomitant use of HAART, during the most important treatment for HCC in HIV-positive patients seems feasible and safe.

## Drug interactions caused by HAART/AC combination

The safeguarding of dose schedule and dose-intensity are the primary goals in treating cancer (Beumer et al., 2014). Several studies have shown that intensive AC protocols are practicable in HIV-patients and the outcome of some of these patients affected with either Burkitt lymphoma or Hodgkin Lymphoma or diffuse large B-cell lymphoma is similar to that of HIV-negative patients receiving the same AC regimens (Ratner et al., 2001). The timing of diagnoses of HIV and a malignancy must guide therapy decisions. In some cases, cancer treatment should take priority over HAART despite the risk related with HIV treatment discontinuation. However, HAART is always recommended, especially if a patient is diagnosed with HIV and malignancy, to prevent the emergence of resistant HIV strains, OI, and death (Beumer et al., 2014; Harrys and Mulanovich, 2014). Nowadays the availability of more than 20 approved antiretrovirals and the possibility of individual genotype profiling, allows the development of protocols that minimize the potential DDIs and improve agreement with HAART during AC (Di Francia et al., 2015a,b). Anthracyclines, antimetabolite agents, antitumor antibiotics, and platinum undergo non-CYP450 routes of elimination and would be unlikely to be altered by HAART. Camptothecins undergo non-enzymatic routes of elimination and are substrates but not inhibitors or inducers of CYP450 and UGT iso-enzymes and, therefore, are likely to be altered by HAART (Harrys and Mulanovich, 2014). Then again, DDIs can be anticipated with alkylating agents, corticosteroids, epipodophyllotoxins, taxanes, tyrosin-kinase inhibitors, and vinca alkaloids (Table 2).

**Table 2:** **Drug–drug interactions in HAART/Antiblastic combined therapy and pharmacogenomics annotations**.

**Antiblastic drug**	**Metabolism**	**Reported interactions**	**^#^Pharmacogenomics annotations**	**Comments**
**TAXANES**				
Paclitaxel	CYP2C8 > CYP3A4	Caution when unboosted atazanavir is coadministered with drugs that are CYP2C8 substrates with narrow therapeutic indices clinically significant interactions with CYP2C8 substrates are not expected when atazanavir is boosted with ritonavir	Breast Cancer Patients carrying CYP2C8*3 haplotype are associated with increased risk of neurotoxicity. Polymorphism T274M in Beta Tubulin VI (BTT VI) gene is associated with severe myelosuppression in patient treated to Taxanes	High taxane levels with CYP3A4 inhibitors may have high risk and severity of myelosuppression and peripheral neuropathy
docetaxel	CYP3A4	Ritonavir		Same to paclitaxel
**VINCA ALKALOIDS**				
Vincristine, vinblastine and vinorelbine	CYP3A4	Ritonavir and others PIs	ND	High vinca levels may have a high risk and severity ofperipheralneuropathy and myelosuppression If possible, consider modifying cART to a non-PI based regimen
**EPIPODOPHYLLOTOXINS**				
Etoposide	CYP3A4 (main); CYP2E1, CYP1A2 (minor)	Risk of toxicity with all CYP3A4 inhibitors	ND	High etoposide/teniposide levels may have high-risk and severity of mucositis, myelosuppression and transaminates
Teniposide				
**ALKYLATING AGENTS**				
Cyclophosphamide	CYP2B6 > 2C19 to active metabolite. CYP3A4 to inactive and possibly toxic metabolites	CYP2B6 inducers (e.g., ritonavir, nelfinavir, efavirenz, nevirapine) and CYP3A4 inhibitors (e.g., PIs, elvitegravir/cobicistat. Etravirine inhibits CYP2C19 Rilpivirine induces CYP2C19; monitor for toxicity	ND	Induction of CYP2B6 has ahigh amount of active metabolite formed. Inhibition of CYP2B6 may prevent activation of the drug. Induction of 3A4 may have neurotoxicity whereas inhibition of CYP3A4 may make more drugs available for 4-hydroxylation route
				Inhibition of CYP2C19 may impact activation of the drug although this may be compensated for by increased shunting through the2B6 pathway
Ifosfamide	CYP3A4 to an active metabolite	May need to hold antiretrovirals or change to a regimen without CYP3A4 inhibitors		CYP3A4 metabolism of (S)-ifosfamide may generate neurotoxic
	CYP3A4 and CYP2B6 involved in detoxification			Induction of CYP3A4 may produce myelosuppression, arrhythmia, hemorrhagic cystitis
Platin-derivates	Primary renal elimination post Glutathione additions (GSTP1, GSTM1, and others)	The potential for pharmacokinetic interactions with ARVs appears minimal. However, cisplatin-induced nephrotoxicity may necessitate dosage adjustment for certain ARVs	Polymorphism *GSTP1* rs1695 Ile105Val (313A>G in exon 5, sometimes labeled GSTP1*B) has been associated with reduced enzyme activity and toxicity	Monitor serum creatinine and creatinine clearance; adjust antiretroviral doses accordingly as needed
		Potential additive renal toxicity with tenofovir		
**ANTHRACYCLINES**				
Daunorubicin	Aldoketoreductase and NADPH-dependent cytochrome reductase	Monitor for efficacy and toxicity with concomitant CYP inhibitors or inducers	Resistance prevention by Detection of MDR1 (ABCB1) 3435C>T (rs1045642)	Potential for interactions unknown, given uncertainty about role of CYP 450 in free radical generation. P-gp inhibitors may increase the intracellular accumulation of doxorubicin, which may enhance cytotoxic effects and/or systemic toxicity
Dactinomycin	Resulting aglycone derivatives conjugated to a sulfate or glucuronide metabolite			
Doxorubicin	Involved in free radical generation. Substrate of P-gp which may influence Intracellular concentrations			
**ANTIMETABOLITES**				
Cytarabine	Metabolized in liver by cytidine deaminase (CDA)	Caution with AZT; tenofovir due to renal toxicity	CDA haplotype: –451C>T, –92A>G, Lys27Gln results in toxicity	Primary toxicities of cytarabine include dose-limiting myelosuppression nausea, vomiting, urinary retention, renal failure (rare)
Fluoropyrimidines	Metabolism by the dihydropyridine dehydrogenase (DPD). 7–20% really excreted. Strong inhibitor ofCYP2C9	Possible interaction with either CyP2C9 inhibitors (eg Efavirenz and Etravirine) or 2C9 Inducer (Elvitegravir)	DPYD*2A haplotype results in severe toxicity	Severe mucositis and gastrointestinal for DPYD deficient
Gemcitabine	extensively metabolized to 2′,2′-difluorodeoxyuridine (dFdU) bY CDA enzyme The main metabolite dFdU has a long terminal half-life after oral administration	Potential for cytochrome-mediated interactions with ARVs appears minimal	Need to assess CDA haplotype: –451C>T, –92A>G, Lys27Gln. In additions check polymorphism on Nucleotide Transporters (hENT1)	Unlikely to result in detrimental pharmacokinetic interactions with cART
**TYROSINE KINASE INHIBITORS**				
Erlotinib	Primarily metabolized by CYP3A4. Metabolized to a lesser extent by CYP1A2 and CYP1A1	dosing reduction of erlotinib 50 mg daily when coadministering with ritonavir 100 mg daily	Erlotinib binding affinity for EGFR exon 19 deletion or exon 21 L858R mutations is higher than its affinity for the wild type receptor	Alternative treatments lacking potent CYP3A4 inducingactivity should be considered when possible
Imatinib	Extensively metabolized by CYP3A4. An N-demethylatedpiperazine. A derivative is the main circulating metabolite	Interferences PIs, NNRTIs, and elvitegravir/cobicistat	Consider specific resistance to imatinib due to acquired mutations of ABL gene (i.e., T315I)	Monitor patients for signs of imatinib dose-related adverse events (fluid retention/weight gain, nausea, and vomiting, neutropenia)
Sunitinib	Metabolized primarily by CYP3A4 to active metabolite SU012662 which is also metabolized by CYP3A4	Avoid concomitant administration of CYP3A4 inhibitors such as PIs and elvitegravir/cobicistat, or inducers such as NNRTIs if possible	Patients with metastatic Renal Carcinoma carrying an ABCG2 421 AA genotype developed significantly more grade 3 or grade 4 thrombocytopenia, neutropenia	Potential for high concentrations of CYP3A4 inhibitors. In healthy volunteers, coadministration of single dose sunitinib and ketoconazole led to 49% high Cmax and 51% high AUC of sunitinib
		Sunitinib dose may be reduced		
**MISCELLANEOUS**				
Irinotecan	SN-38 metabolite (active); CYP3A4 and Glucuronidation by UGT1A1	Potential for augment irinotecan-related toxicities with atazanavir, which also inhibits UGT1A1	Need to detect UGT1A1 *28. Haplotype carrying TA repeat 7/7 is high-risk toxicity due poor metabolizer	Inhibition of 3A4 may have a high risk and severity of myelosuppression. Induction of 3A4 or glucuronidation may augment the efficacy of the drug
Tamoxifen and Aromatase Inhibitors	Multiple isoenzymes involved: CYP3A4 > CYP1A2 to N-desmethyltamoxifen. In addition CYP2D6, 2C9, 2C19, 3A4, and 2B6 to trans-4-hydroxytamoxifen. May induce to CYP3A4	The potential for reduction levels of PIs, NNRTIs or elvitegravir/cobicistat	Genotyping FDA and EMA recommendation guidelines for CYP2D6*4 (Pro34Ser)	Inhibition of 3A4 may augment risk and severity of tamoxifen related side effects (e.g., hot flushes, nausea, and vomiting)
				Avoid concomitant use of CYP2D6 inhibitors
Exemestrane Letrozole	Metabolized by CYP3A4 and Aldoketoreductases Letrozole is a substrate to CYP2A6 too	Nevirapine and efavirenz may reduce efficacy. High levels with PIs and delavirdine may augment risk and severity of adverse effects (e.g., musculoskeletal pain, peripheral edema, hot flashes, etc.	Genome-wide study in breast cancer treated with aromatase inhibitors shown significant polymorphism in Tubulin Beta 1 (TUBB1) (rs10485828)	Avoid combination to efavirenz and PIs if possible
***Corticosteroids*** Dexamethasone Prednisone	CYP3A4 Dexamethasone is a CYP3A4 inducer	Dexamethasone may reduce levels of NNRTIs, PIs, and elvitegravir/cobicistat	Resistance prevention by Detection 3435C>T (rs1045642). In addition check Vitamin D receptor (VDR) *Taq, Apa, BsmI, FokI*	Consider the use of non-CYP3A4 inducing steroid, or modifying to a non-CYP based cART regimen (e.g., dolutegravir, raltegravir)
Bortezomib	Metabolized primarily by CYP3A4, CYP2C19, CYP1A2, CYP2D6, and CYP2C9 to a minor extent. It may inhibit CYP2C19 at clinically relevant dosages	Efavirenz and etravirine inhibit CYP2C19 and induce CYP3A4. Clinical significance unknown; monitor for bortezomib efficacy and toxicity. Rilpivirine induces CYP2C19		Potential variation for bortezomib concentrations with potent CYP inhibitors or inducers of CYP3A4 and CYP2C19. monitor for efficacy

# Referred to Pharmacogenomics Knowledge Base www.pharmgkb.com Source: http://www.hiv-druginteractions.org/data/ExtraPrintableCharts/ExtraPrintableChartID7.pdf

### Taxanes

Several trials have established the efficacy of paclitaxel for the treatment of AIDS-related KS. Concomitant administration of paclitaxel with CYP3A4 inhibitor causes an increase in taxane concentrations with an increased risk of severe myelosuppression and peripheral neuropathy (Leandro-García et al., 2012). The CYP3A4 inducers dexamethasone and efavirenz do not have an important effect on docetaxel exposure (Rudek et al., 2014). In an *in vivo* experiment, docetaxel 20 mg/kg IV was administered in the both concomitant and absence of dexamethasone or efavirenz for 4 days, or single dose ketoconazole or ritonavir. The CYP3A4 inducers efavirenz and dexamethasone did not have a noteworthy effect on docetaxel AUC. Nevertheless, the CYP3A4 inhibitors ritonavir and ketoconazole resulted in a 6.9- and a 3.1-fold increase in docetaxel AUC, respectively (Rudek et al., 2014).

### Vinca alkaloids (vinblastine, vincristine, vinorelbine)

The vinca alkaloids remain an important class of AC traditionally used in the treatment of breast, lung, testicular cancer and currently (Vinorelbine, Vinblastine) also in the management of AIDS-related KS. Vinca alkaloids are substrates of CYP3A4 and are susceptible to PI and NNRTI. Concomitant administration with CYP3A4 inhibitors antagonizes vinca alkaloids metabolism with an increased risk of neurotoxicity and severe myelosuppression. Interaction between ritonavir/lopinavir and vincristine is mainly responsible for paralytic ileus. In fact, vincristine is transferred by P-gp and is metabolized by CYP3A4. Ritonavir is a potent CYP3A4 and P-gp inhibitor. Lopinavir is also a P-gp inhibitor. These PIs can induce a delayed vincristine elimination (Kohlrausch et al., 2010). Conversely, CYP3A4 inducers cause decrease of vinca alkaloids concentrations with decreased efficacy of drugs. In addition, the impact of SNPs on the drug transporter *SLCO1B1* is related to plasma levels of Lopinavir and Ritonavir (Kohlrausch et al., 2010).

### Epipodophyllotoxins (etoposide and teniposide)

This class of AC is primarily used for the management of hematological malignancies. Their metabolism is mediated by the CYP3A4 pathway; therefore, inhibition of the CYP3A4 pathway can increase blood concentrations of epipodophyllotoxins with an increased risk of mucositis, liver toxicity and myelosuppression (Antoniou and Tseng, 2005; Beumer et al., 2014).

### Alkylating agents (cyclophosphamide and ifosfamide)

Despite their structural similarity and similar mechanisms of action, significant differences exist in the metabolism of cyclophosphamide and its isomer ifosfamide. Cyclophosphamide is an alkylating agent used in the management of HD and NHL for patients with HIV and is metabolized by two separate pathways (CYP3A4 and CYP2B6). Induction of CYP2B6 may increase the amount of active formed metabolite; conversely PI may decrease the efficacy of cyclophosphamide through CYP2B6 inhibition. A pharmacokinetic analysis conducted in 29 HIV-positive patients with NHL treated with CHOP with and without concomitant indinavir showed a reduce of cyclophosphamide clearance from 70 to 41–46 mL/min/m^2^. This, didn't translate into extreme toxicity (Spina and Tirelli, 2004). Induction of CYP3A4 could make more drug available for the 4-hydroxylation route and increase the efficacy and toxicity of cyclophosphamide. In contrast, ifosfamide is administered as a racemic mixture of its two enantiomeric forms: R and S-ifosfamide, metabolized through the CYP3A4 pathway. Induction of CYP3A4 can increase activation of the drug and can also generate more potentially neurotoxic metabolite (Wainer et al., 1994; Antoniou and Tseng, 2005).

### Anthracyclines (daunorubicin and doxorubicin)

Anthracyclines are regularly used agents in the treatment of both AIDS-related NHL and KS. Luckily, the potential for DDIs between CYP-pathways and anthracyclines appears to be minimum. Interactions with PIs or NNRTIs and CYP-pathways may decrease reduction of free radicals, which may decrease both antineoplastic and cytotoxic properties of the AC agents. Enzyme inducers can do the opposite. Two pharmacokinetic analyses were performed in HIV-patients with NHL treated with CHOP with and without concurrent PI-based HAART (Vaccher et al., 2001; Spina and Tirelli, 2004). The earliest study in 19 patients showed that doxorubicin pharmacokinetics was not affected by simultaneous PI administration, and PI exposures were not altered by doxorubicin (Ratner et al., 2001).

### Antimetabolites

They include several nucleoside analog drugs used in combination with others antineoplastics in carcinomas and NHLs. Luckily, the potential for adverse drug interactions with HAART appears to be minimal, but the clinical trials in this field are small.

Potential toxicity is considered for high exposures to etravirine due to CYP2C9 inhibition. However, close monitoring should be considered.

A cohort of 21 HIV-patients treated with HAART (seven NRTI only, six on PI, six on NNRTI, and two on PI/NNRTI-containing regimens) developed anal carcinoma and received radiotherapy plus MMC and 5-FU without the need for dose reductions. The CR rate was 81%, and 62% remained free of any tumor relapse during additional follow-up (median, 53 months), without increased risk of HIV progression (Fraunholz et al., 2010).

Case series of five HIV-positive patients on HAART (4 PI, 1 NRTI) with advanced colorectal cancer received oxaliplatin, leucovorin and fluorouracil (FOLFOX-4 regimen) without apparent increase in antineoplastic associated toxicity (Berretta et al., 2008).

### Miscellaneous

#### Camptothecins

Irinotecan (CPT-11), is a DNA topoisomerase I inhibitor with a broad range of activity against solid tumors. The model of (beta Fibrinogen Growth Factor) bFGF-induced angiogenesis in mouse cornea suggested that Irinotecan is also active in HIV-related KS (Martellotta et al., 2009). Recent data show that lopinavir/ritonavir has a strong effect on the pharmacokinetic profile of CPT-11 when used alone in HIV patients with advanced KS (Corona et al., 2008). Lopinavir/ritonavir reduces the clearance of CPT-11 by 47%; the oxidized metabolite APC inhibited the formation of SN38 glucuronide catabolite by 81%. This effect resulted in increased availability of blood SN38 active metabolite with consequent increased severe toxicity. Conversely, induction of CYP3A4 or glucuronidation can decrease the effectiveness of the drug (Corona et al., 2008). Pharmacogenomics profile UGT1A1 *28 haplotype with homozygous 7 TA repeat are high risk for irinotecan-related toxicities with atazanavir, which also inhibits UGT1A1 (Corona et al., 2008).

#### Tamoxifen

It is commonly used as an estrogens antagonist and undergoes wide hepatic metabolism involving some isoforms of the Cytochromes. Induction of CYP3A4 by tamoxifen may decrease NNRTI or PI concentrations. Conversely, inhibition of CYP3A4 isoforms with PIs or NNRTIs can increase efficacy and both risk and severity of tamoxifen-related adverse effects. Numerous studies have shown that nelfinavir promotes in cancer cells, autophagy and apoptosis cell cycle arrest, and may be a valuable drug against breast cancer when combined with tamoxifen in patients with hormone-responsive tumors (Brüning et al., 2010). Interactions between HAART and aromatase inhibitors are also hypothetically practicable (Liu et al., 2014). Letrozole and exemestrane are both metabolized by CYP3A4 to inactive metabolites. NNRTIs may decrease the efficacy of drugs conversely PIs may increase concentration and severity of adverse effects of both letrozole and exemestrane (Buzdar et al., 2002).

#### Corticosteroids

Corticosteroids, are part of combination AC regimens and may be subject to changes in their pharmacokinetic and pharmacodynamic effects as a result of antiretroviral-mediated modulation of their biotransformation. In particular dexamethasone and methyl prednisolone are vulnerable to interactions with HAART since the CYP3A4 isoform is the primary enzyme mediating the metabolism of these drugs. Dexamethasone may decrease concentrations of NNRTIs and PIs. PIs may increase the pharmacodynamic effects of corticosteroids when used concurrently. Conversely, CYP3A4 inducers may reduce the efficacy of these drugs. Therefore, it is necessary to stop HAART in patients receiving prolonged dexamethasone or alternatively consider the use of non-CYP3A4 inducing corticosteroid or antiretroviral drugs, with monitoring if the combination is necessary (Antoniou and Tseng, 2005; Mounier et al., 2009).

#### Tyrosine kinase inhibitors

Tyrosine kinase inhibitors account for a large panel of small molecules that target tyrosine kinase protein domains of the wide range species of growth factors receptors.

Erlotinib is approved for the treatment of non-small cell lung (NSCLC) and pancreatic cancer. It is metabolized by CYP3A4. Inducers or inhibitors of CYP3A4 enzymes such as PIs (e.g., ritonavir) or NNRTIs (e.g., efavirenz) can modify the metabolism and efficacy of the drug. Recent data propose that to get the desired drug exposure, the clinically used dose (150 mg daily) of Erlotinib must be significantly reduced (25 mg every day) or increase (300 mg daily), respectively, when ritonavir or efavirenz are co administered (Pillai et al., 2013).

Imatinib, a specific inhibitor of a tyrosine kinase associated with the proto-oncogene c-kit, used in the treatment of chronic myelogenous leukemia, is also metabolized by the CYP450 system.

Sunitinib, an oral multi-targeted tyrosine kinase inhibitor used for the treatment of advanced renal cancer and gastrointestinal stromal tumors (GISTs) is biotransformed by CYP3A4 into a most important pharmacologically active N-desmethyl metabolite (Mounier et al., 2009). The inhibition of proteasomal activity by specific proteasome inhibitors or cross-reactivity of particular PIs with proteasomal enzymes recently became of interest because of the anti-tumor properties of these molecules.

#### Bortezomib

Bortezomib used in association with nelfinavir, it induces cell cycle arrest in cervical cancer cells as reflected by marked changes in the expression of cell cycle regulatory cyclins and ensuring mitochondrial independent apoptosis (Bruning et al., 2001). Therefore, the combination with ritonavir inhibits renal cancer growth synergistically at clinically feasible concentrations. The effectiveness of the combination is caused by protein ubiquitination and histone acetylation. In urological cancers, Bortezomib was used in combination with Ritonavir with a positive effect on protein ubiquitination (Bibas et al., 2010; Sato et al., 2012).

#### Lenalidomide

Lenalidomide, is an analog of thalidomide that does not show pharmacokinetic interaction with HAART because it is not metabolized by the liver but is eliminated by the renal route (Hertz et al., 2012).

## Conclusion

As patients with HIV live longer, they develop more malignancies that are both HIV-related and unrelated. On this basis, a better understanding of AC and HAART interactions is urgently needed. DDIs are frequently encountered in the therapy of cancer patients with HIV. All PIs are inhibitors of CYP3A, which is central route in the metabolism of ~50% of all AC drugs. Among the PIs, ritonavir is the strongest inhibitor of CYP3A activity. Conversely, NNRTIs can induce metabolism and potentially reduce the efficacy of AC drugs. Even though raltegravir has little potential for DDIs, the occurrence of viral mutations limits its use as a single molecule. Interactions may also be a consequence of a modification of the activities of glucuronosyltransferase and/or of transport proteins. Ritonavir is an inhibitor of P-glycoprotein, which leads to increased exposure to many AC drugs. Generally, to prevent DDIs and avoid severe toxicity, treatment options include substitution of an antiretroviral alternative or temporary discontinuation of HAART or selection of an alternative chemotherapy regimen. Zidovudine is linked with severe neutropenia hence it should not be combined with cytotoxic regimens containing neutropenic agents. Didanosine and stavudine, old generation NRTIs, are related with irreversible peripheral neuropathy which is also a common side effect of platinating agents, taxanes, vinca alkaloids, and bortezomib. AC-induced neuropathy is generally cumulative or dose-related with management consisting of dose-reduction or lower dose intensity. PIs and newer molecularly targeted anti-cancer agents including the tyrosine kinase inhibitors can cause QT prolongation, arrhythmias, and sudden death. In addition, PIs appear to significantly potentiate the myelotoxicity of AC. Bilirubin is often used as a marker for dose adjustment for AC agents such as docetaxel, doxorubicin, etoposide, irinotecan, paclitaxel, sorafenib, and vincristine. Several antiretrovirals such as atazanavir and indinavir are associated with unconjugated hyperbilirubinemia secondary to UGT1A1 inhibition comparable to that which occurs in Gilbert's syndrome. If no other signs of liver dysfunction exist, suggested dose modifications of AC based on liver function tests can be ignored. Therefore, it is important that patients with cancer should be screened for HIV infection, and treatment of HIV infection should be started immediately. HAART should be individualized according to the cancer treatment plan (AC or radiotherapy or surgery), liver or renal diseases, bone marrow suppression, mitochondrial dysfunction and individual patient genotype.

Currently, drug interaction based on individual genomic profile allows the prediction of the toxicity/inefficacy of HAART/AC combined therapy (Table 2). It is well-known that the response of taxane-based therapy is dependent on the individual' CYP2C8*3 allele and β-tubulin VI genotype profile (Di Francia et al., 2013). The neurological effect of FOLFOX therapy could be predicted by a pharmacogenomic panel test performed before therapy (Lamba, 2009), as well as the lethal effect of the DPYD risk genetic variant (Catapano et al., 2014). In addition, therapy based on cytarabine and its related drug Gemcitabine are affected by several polymorphisms found in the Cytidine Deaminase (CDA) gene (Mitra et al., 2012; Kim et al., 2013).

A change in the HAART regimen should be considered in the case of overlapping toxic effects or DDIs between antiretroviral drugs and AC or other drugs or to improve adherence and tolerability.

We are now seeing that HIV treatment has entered into new era in which multidrug treatments and genetic variations (host and virus) must be taken into consideration when planning chemotherapeutic/HAART regimens, in order to maximize benefits and minimize toxicity (Gross et al., 2014).

Finally, a standard prophylaxis against OIs should be tailored with drugs required for specific AC regimens.

In this scenario, the importance of cooperation between oncologists and other health specialist (i.e., infectious disease, pharmacogenetic, and lab specialist) must not be underestimated in the management of these patients and design of an adequate treatment strategy.

## Author contributions

MB, MC, UT, and RD design the study; FM, SZ, AL, CF, LA, TM, and DV research the bibliography. MB, RD wrote the paper.

### Conflict of interest statement

The authors declare that the research was conducted in the absence of any commercial or financial relationships that could be construed as a potential conflict of interest.
